# Antibodies against Platelet Glycoproteins in Clinically Suspected VITT Patients

**DOI:** 10.3390/antib13020035

**Published:** 2024-05-01

**Authors:** Romy T. Meier, Leendert Porcelijn, Suzanne Hofstede-van Egmond, Camila Caram-Deelder, Jonathan M. Coutinho, Yvonne M. C. Henskens, Marieke J. H. A. Kruip, An K. Stroobants, Jaap J. Zwaginga, C. Ellen van der Schoot, Masja de Haas, Rick Kapur

**Affiliations:** 1 Department of Experimental Immunohematology, Sanquin Research and Landsteiner Laboratory, Amsterdam UMC, University of Amsterdam, 1066 CX Amsterdam, The Netherlands; r.meier@sanquin.nl (R.T.M.); e.vanderschoot@sanquin.nl (C.E.v.d.S.); 2 Sanquin Diagnostic Services, Department of Immunohematology Diagnostics, Sanquin, 1066 CX Amsterdam, The Netherlands; l.porcelijn@sanquin.nl (L.P.); s.vanegmond@sanquin.nl (S.H.-v.E.); m.dehaas@sanquin.nl (M.d.H.); 3 Department of Clinical Epidemiology, Leiden University Medical Center, 2333 ZA Leiden, The Netherlands; c.caram-deelder@lumc.nl; 4 Department of Neurology, Amsterdam UMC, 1105 AZ Amsterdam, The Netherlands; j.coutinho@amsterdamumc.nl; 5 Central Diagnostic Laboratory, Maastricht University Medical Centre+, 6229 HX Maastricht, The Netherlands; yvonne.henskens@mumc.nl; 6 Department of Haematology, Erasmus MC, Erasmus University Medical Center, 3015 CN Rotterdam, The Netherlands; m.kruip@erasmusmc.nl; 7 Department of Clinical Chemistry, Radboud University Medical Center, 6525 GA Nijmegen, The Netherlands; an.stroobants@radboudumc.nl; 8 Department of Hematology, Leiden University Medical Center, 2333 ZA Leiden, The Netherlands; j.j.zwaginga@lumc.nl

**Keywords:** platelet-autoantibodies, thrombocytopenia, thrombosis, COVID-19, vaccination

## Abstract

Vaccine-induced thrombotic thrombocytopenia (VITT) is a rare but severe complication following COVID-19 vaccination, marked by thrombocytopenia and thrombosis. Analogous to heparin-induced thrombocytopenia (HIT), VITT shares similarities in anti-platelet factor 4 (PF4) IgG-mediated platelet activation via the FcγRIIa. To investigate the involvement of platelet-antibodies in VITT, we analyzed the presence of platelet-antibodies directed against glycoproteins (GP)IIb/IIIa, GPV and GPIb/IX in the serum of 232 clinically suspected VITT patients determined based on (suspicion of) occurrence of thrombocytopenia and/or thrombosis in relation to COVID-19 vaccination. We found that 19% of clinically suspected VITT patients tested positive for anti-platelet GPs: 39%, 32% and 86% patients tested positive for GPIIb/IIIa, GPV and GPIb/IX, respectively. No HIT-like VITT patients (with thrombocytopenia and thrombosis) tested positive for platelet-antibodies. Therefore, it seems unlikely that platelet-antibodies play a role in HIT-like anti-PF4-mediated VITT. Platelet-antibodies were predominantly associated with the occurrence of thrombocytopenia. We found no association between the type of vaccination (adenoviral vector vaccine versus mRNA vaccine) or different vaccines (ChAdOx1 nCoV-19, Ad26.COV2.S, mRNA-1273, BTN162b2) and the development of platelet-antibodies. It is essential to conduct more research on the pathophysiology of VITT, to improve diagnostic approaches and identify preventive and therapeutic strategies.

## 1. Introduction

Vaccine-induced thrombotic thrombocytopenia (VITT) is a disorder that has been recognized since the global vaccination strategy against SARS-CoV-2 started [1,2]. VITT was initially characterized by thrombocytopenia and thrombosis, and shows similarities with heparin-induced thrombocytopenia (HIT) in terms of clinical characteristics and underlying mechanism [3,4]. In HIT, antibodies are directed against platelet factor 4 (PF4)/heparin complexes resulting in FcγRIIa-dependent platelet activation, while in VITT PF4-antibodies have been identified [1]. Interestingly, besides the more recognized role for PF4-antibodies, a possible role for antibodies against platelet membrane glycoproteins (GPs) has recently been suggested [5]. Platelet-autoantibodies have been implicated in diseases including sepsis and the autoimmune disorder immune thrombocytopenia (ITP), in which platelet clearance is mediated by platelet-autoantibodies [6]. In addition, platelet-associated IgG was shown to be elevated in thrombocytopenic patients with sepsis [7]. Whereas healthy individuals generally do not test positive for platelet antibodies in the MAIPA, 18% of ITP patients test positive for GPV, 15% for GPIIb/IIIa and 15% for GPIb/IX in the indirect MAIPA [8,9]. Given the role of platelet-autoantibodies in thrombocytopenia, it is possible these platelet-autoantibodies play a role in the pathophysiology of VITT.

A study found that healthy recipients of both adenoviral vector and mRNA vaccines developed platelet-autoantibodies without a clear preference for one of the tested platelet glycoproteins (GP) IIb/IIIa, Ib/IX and Ia/IIa [10]. In another study, 30% of the 27 proven VITT patients vaccinated with ChAdOx1 nCov-19 tested positive for free-circulating platelet-antibodies targeting platelet GPIIb/IIIa, GPIb/IX or GPIa/IIa [5]. To gain more insight into the significance of antibodies against platelet glycoproteins, we conducted an analysis in all known clinically suspected VITT individuals determined by physicians based on the (suspicion of) occurrence of thrombocytopenia/thrombosis upon COVID-19 vaccination in the Netherlands.

## 2. Materials and Methods

We tested clinically suspected VITT patients for the presence of platelet-antibodies. Due to lack of availability of patient platelets, we used an indirect monoclonal antibody immobilization of platelet antigens (MAIPA) assay [11]. This assay is considered the gold standard reference technique in platelet immunology and is used in the Netherlands to support the diagnosis of immune thrombocytopenia (ITP) [11,12]. The MAIPA was performed as described by Kiefel et al. [11], in brief: microtiter plates were coated with goat-anti-mouse (GαM) for 12 h at 4 °C. Following this, platelets were washed and patient serum was added to the plate. Subsequently, monoclonal antibodies directed against circulating antibodies (GPIIb/IIIa (αIIbβ3, CD41/CD61, CLB/Thromb1 (C17), Sanquin Reagents), GPV (CD42d, SW16, Sanquin Reagents) and GPIb/IX (CD42c/CD42a, FMC25, ThermoFisher)) were introduced [8]. After washing and centrifugation, a GαM–HRP conjugate was added to the plate. After further washing, extinction was measured using an ELISA reader (Epoch ELISA reader). An extinction of ≥0.130 was interpreted as positive, while an extinction of ≤0.130 was regarded as negative.

Furthermore, we measured free circulating plasma thrombopoietin (TPO) levels to gain insights into platelet production or platelet breakdown. TPO levels were measured in EDTA-anticoagulated plasma samples using an in-house-developed TPO sandwich ELISA, as described by Folman et al. [13]: microtiter plates were coated with two non-cross-reactive monoclonal antibodies. After washing and blocking the plates, a third biotinylated monoclonal antibody and patient plasma were added. Following further washing, a streptavidin–horseradish–peroxidase was added and H_2_SO_4_ was added to stop the reaction. The extinction was determined using an ELISA reader (Epoch ELISA reader). Results were reported as “normal” (0–60 U/mL plasma) and “elevated” (>60 U/mL plasma).

Since D-dimer data were missing at the time that the samples were collected, we were unable to adhere to the later and currently established VITT classification [14,15]. We therefore categorized clinically suspected VITT patients based on the occurrence of thrombocytopenia and/or thrombosis. For VITT diagnostic testing we used an in-house-developed anti-PF4 in which patient serum was added to a PF4-coated (Chromatec, Greifswald, Germany) microtiter plate. PF4-antibodies were detected measuring excitation after adding GaH-HRP IgG to the plate. Patients with an OD ≥ 1.0 were considered positive. In the PIPAA, performed as described by Greinacher et al. [1] with slight modifications, we incubated washed donor platelets with PF4 and with and without FcγRIIa (CD32)-blocking monoclonal antibody clone IV.3 (Sanquin Research, Amsterdam, The Netherlands). Patients with both thrombocytopenia and thrombosis, and testing positive in both diagnostic tests, were classified as HIT-like VITT patients. This classification aligns with the confirmation criteria for HIT patients, who are identified by a positive anti-heparin/PF4-ELISA and a positive FcγRIIa-dependent heparin-induced platelet activation assay (HIPAA) [16,17].

To estimate the incidence of platelet-antibodies in COVID-19-vaccinated individuals we used data on the total number of vaccines within our study period which was obtained from the National Institute for Public Health and the Environment (RIVM) and encompasses all COVID-19 vaccination data within The Netherlands.

## 3. Results

### 3.1. Patient Characteristics

We examined 232 patients clinically suspected of VITT, for whom we received samples for diagnostic testing between 22 March and 26 November 2021 (Table 1). Our cohort consisted of 111 females and 121 males with a median age of 62 (IQR: 53–68). Of the 232 VITT suspected patients 112 (48%) were vaccinated with ChAdOx1 nCoV-19, seven (3%) with Ad26.COV2.S, 34 (15%) with mRNA-1273, and 79 (34%) with BTN162b2. Patients were admitted, on average, 21 days after vaccination.

Our cohort contained seven confirmed HIT-like VITT patients (for patients’ description: Table S1). All other patients tested negative in both the anti-PF4 IgG ELISA and FcγRIIa-dependent PIPAA or did not have both thrombocytopenia and thrombosis (for patients’ description: Table S2).

### 3.2. Platelet-Antibodies in HIT-like VITT Patients

**Figure 1:**
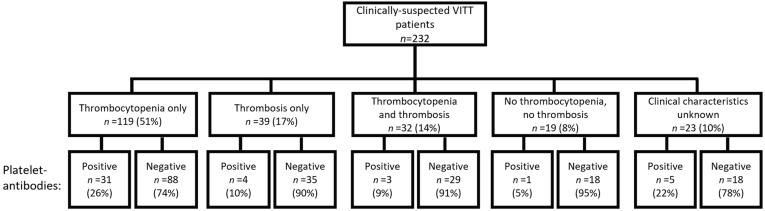
Anti-platelet GP in clinically suspected VITT patients after vaccination with ChAdOx1 nCoV-19, BNT162b2, mRNA-1273 or Ad26.COV2.S. Serum samples of 232 unique and clinically suspected VITT patients were analyzed for the presence of platelet-autoantibodies.

We did not observe platelet-antibodies in HIT-like VITT patients (*n* = 7). However, we found that 44 clinically suspected VITT patients in our cohort tested positive for platelet-antibodies; 26% (*n* = 31) of patients with isolated thrombocytopenia (platelet count <100 × 10^9^/L), 10% (*n* = 4) of patients with thrombosis only, 9% (*n* = 3) of patients with both thrombocytopenia and thrombosis, and 5% (*n* = 1) of patients with neither thrombocytopenia nor thrombosis (Figure 1).

### 3.3. Clinical Characteristics in Clinically-Suspected VITT Patients with Platelet-Antibodies

Within the 44 platelet-antibody positive patients, we observed a higher incidence of thrombocytopenia (77%), compared to the group testing negative for platelet-antibodies (62%) (Table 1). Remarkably, a smaller proportion of the platelet-antibody positive group (16%) presented with thrombosis, compared to the platelet-antibody negative group (34%). The combination of thrombocytopenia and thrombosis was less common in patients positive for platelet-antibodies. It should be noted that data on thrombocytopenia and/or thrombosis were not available for all patients, and these patients were not included in this analyses.

### 3.4. Presence of Platelet-Antibodies in Relation to Vaccines

In our cohort, 17% (*n* = 19) of ChAdOx1 nCov-19 vaccinees, 22% (*n* = 17) of BNT162b2 vaccinees, 21% (*n* = 7) of mRNA-1273 vaccinees and 14% (*n* = 1) Ad26.COV2.S vaccinees tested positive for platelet-antibodies (Table 1). Within this cohort, 20 patients vaccinated with adenoviral vector vaccines tested positive for platelet-antibodies out of a total 3,304,944 doses given nationwide during the study period (0.61 cases per 100,000 adenoviral vector-based COVID-19 vaccine doses). Additionally, 24 patients vaccinated with mRNA-based vaccines tested positive for platelet-antibodies out of a total of 20,670,060 given doses (0.12 cases per 100,000 mRNA-based COVID-19 vaccine doses).

**Figure 2:**
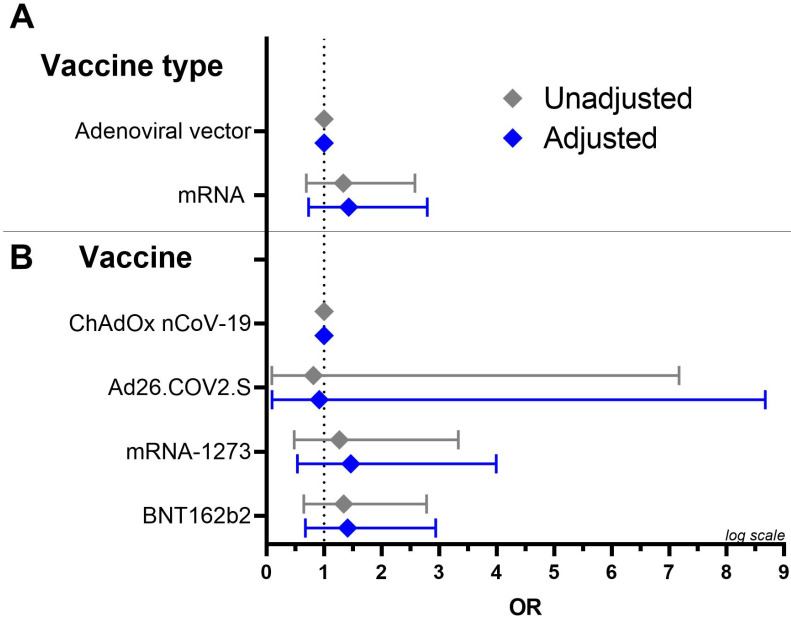
Forest plot for odds ratios with 95% CI for the effect on presence of platelet-antibodies. We corrected for age (continuous) and sex (female vs. male). (**A**) mRNA vaccines were compared with adenoviral vector vaccines (baseline). (**B**) BNT162b2, mRNA-1273 and Ad26.COV2.S were compared to ChAdOx1 nCoV-19 (baseline).

To determine whether there was a relationship between the presence of platelet-antibodies and the type of vaccine (adenoviral vector vaccine vs. mRNA vaccine) we performed a multivariate logistic regression to determine the effects of age and sex on the likelihood that clinically suspected VITT patients vaccinated with adenoviral vector vaccines will develop platelet-antibodies versus suspected VITT patients vaccinated with mRNA vaccines (Figure 2, panel A). We found no difference in the risk of developing platelet-antibodies between being vaccinated with the adenoviral vector and the mRNA vaccine (OR = 1.43, 95% CI [0.73; 2.79]) as the logistic regression model was not significant (*p*-value = 0.465) and explained 1.1% (pseudo R^2^) of the variance of the presence of platelet-antibodies.

We performed a similar analysis to investigate the relationship between the presence of platelet-antibodies and the four different vaccines (Figure 2, panel B). With ChAdOx1 nCov-19 as our reference, we found no difference in risk of developing platelet-antibodies between patients vaccinated with the four different vaccines; the BNT162b2 vaccine (OR = 0.92, 95% CI [0.10; 8.7]), the mRNA-1273 vaccine (OR = 1.46, 95% CI [0.54; 4.0]) and the Ad26.COV2.S vaccine (OR = 1.41, 95% CI [0.68; 2.94]). The logistic regression model was not significant (*p*-value = 0.766) and explained 1.1% (pseudo R^2^) of the variance of the presence of platelet-antibodies. However, it is important to note that in this analysis the small group size and poor model performance (small pseudo R^2^) diminishes the power of detecting a possible relevant and significant change.

### 3.5. Platelet-Antibody Profiles

**Figure 3:**
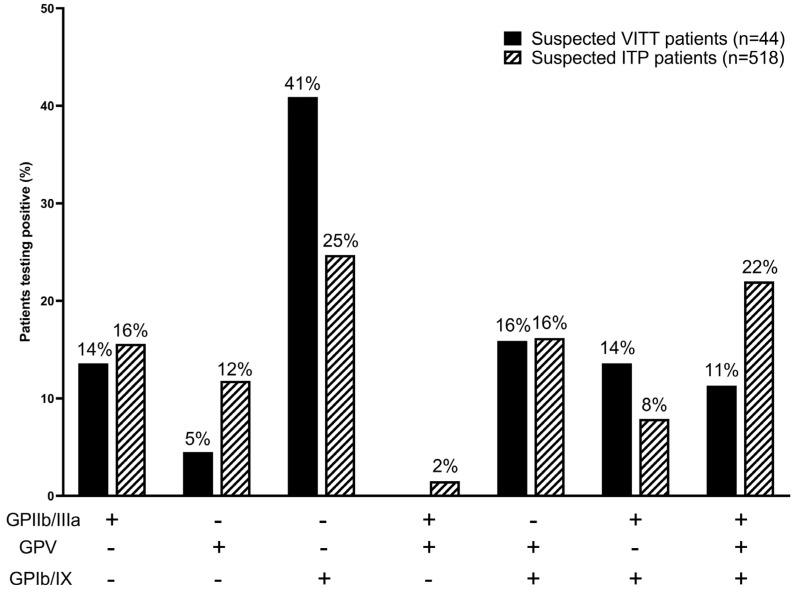
Percentage of suspected VITT or ITP patients (Y-axis) positive for glycoprotein specific platelet-antibodies (X-axis). Solid bars are suspected VITT patients, dashed bars are suspected ITP patients. Glycoprotein-specific anti-platelet (GPIIb/IIIa, GPV, GPIb/IX) detection stratified to type of vaccine in clinically suspected VITT (*n* = 44) and suspected ITP (*n* = 518) were not significantly different (X-squared = 10.592, df = 6, *p*-value = 0.1018).

To further investigate whether the platelet-antibody positive patients in our cohort were ITP patients, we compared antibody profiles of suspected VITT patients with antibody profiles of suspected ITP patients. Out of the 44 suspected VITT patients positive for platelet-antibodies, 14% tested positive for GPIIb/IIIa, 5% for GPV, 41% for GPIb/IX-antibodies and 11% tested positive for all three platelet-antibodies (Figure 3). In comparison, of patients tested in the MAIPA in our institute in the years 2022 and 2023 due to suspected ITP, 518 out of 1507 (34%) patients tested positive for platelet-antibodies; 16% for GPIIb/IIIa, 12% for GPV, 25% for GPIb/IX, and 22% tested positive for all three platelet-antibodies. Although we found that anti-GPIb/IX antibodies were increased in clinically suspected VITT patients (41%) vs. in suspected ITP patients (25%), overall antibody profiles between clinically suspected VITT patients and suspected ITP patients were not statistically significant (X-squared = 10.592, df = 6, *p*-value = 0.1018).

### 3.6. TPO Levels of Clinically-Suspected VITT Patients

We examined the levels of thrombopoietin (TPO) in the plasma of 42 patients to determine the probability of identifying patients positive for platelet antibodies as ITP patients, in which TPO levels are normal/non-elevated [18,19]. We determined the TPO levels of 42 of 44 platelet-antibody positive patients and 178 platelet-antibody negative patients, of which seven HIT-like VITT patients. Out of the seven HIT-like VITT patients, two (29%) patients had high TPO levels and five (71%) patients had normal TPO levels. Out the 42 patients testing positive for platelet-antibodies, the majority of 25 (59%) patients with normal TPO levels, and four (10%) patients with elevated TPO levels presented with thrombocytopenia (Figure S1). Since ITP patients generally do not have elevated TPO levels, we cannot rule out that patients in our cohort with normal TPO levels are ITP patients.

## 4. Discussion

In our investigation into the potential role for platelet-autoantibodies in VITT pathophysiology, we analyzed the presence of platelet-antibodies in a cohort of 232 clinically suspected VITT patients, including seven HIT-like VITT patients. We did not detect circulating platelet-autoantibodies in HIT-like VITT patients, implying that platelet-autoantibodies may not be involved in the pathophysiology of HIT-like VITT. Interestingly, three out of seven HIT-like VITT patients (43%) were diagnosed with intracranial thrombosis which is found to be a hallmark for VITT (Table S1) [20]. We found that 44 patients (19%) in our cohort of clinically suspected VITT patients tested positive for platelet-antibodies. These platelet-antibodies were predominantly detected in patients with thrombocytopenia, raising the possibility of a mechanism of antibody-mediated platelet clearance. It therefore seems likely that other platelet-antibody-independent mechanisms may underlie the development of thrombosis (with or without thrombocytopenia) in VITT patients. Analysis of platelet-antibody levels in the non-thrombocytopenic and COVID-19-vaccinated control group would be required in order to study this further, but this group was unfortunately not available to us.

Considering platelet-autoantibodies have been found in both adenoviral vector and mRNA COVID-19 vaccine recipients [21,22,23], but not healthy individuals [8,24], we examined the association between the (type of) vaccine(s) and the presence of platelet-autoantibodies. We found that the risk of developing antibodies was independent of the (type of) vaccine and we therefore concluded there is no association between the (type of) vaccine or the presence of platelet-antibodies in clinically suspected VITT patients. Thus, it remains unclear what may have caused the presence of these platelet-antibodies in clinically suspected and non-HIT-like VITT patients.

Since testing for platelet-autoantibodies is generally performed to support an ITP diagnosis, it is plausible that some of the patients testing positive for the platelet-antibodies could be (de novo/pre-existing) ITP patients. Since data on underlying conditions in patients are not available to us, we explored whether these patients could be ITP patients; we analyzed the platelet-autoantibody profile in our cohort of clinically suspected VITT patients and compared it to those of ITP patients (Figure 2). Although we did not find overall differences in antibody profiles between suspected VITT patients and suspected ITP patients, we did find that 41% of the 44 suspected VITT patients positive for platelet-antibodies tested positive for antibodies directed against GPIb/IX. This discrepancy suggests that vaccination could result in the production of platelet-autoantibodies with a preference for epitopes located on platelet-GPIb/IX.

Furthermore, we analyzed TPO levels in patient plasma to further determine the likelihood of platelet-antibody positive patients being classified as ITP patients, which in ITP patients generally demonstrate normal/non-significantly elevated TPO levels [18,19]. TPO, a protein produced mainly in the liver and secreted into the circulation, is the main regulator of thrombopoiesis and can bind to TPO receptors on circulating platelets and megakaryocytes and megakaryocyte precursors [25]. Circulating TPO is primarily cleared by platelets through binding to the TPO receptor followed by internalization and consumption of TPO. Although TPO levels in the blood and bone marrow are inversely related to platelet count, high TPO levels are more likely to indicate an issue in the production of platelets [18,19]. Considering that ITP patients commonly show normal or slightly elevated TPO levels, the 25 (59%) patients with thrombocytopenia who tested positive for platelet-antibodies and had normal TPO levels, might be ITP cases. However, taking into account that ITP is diagnosed through the exclusion of other conditions, and follow-up data are missing, further clinical information is necessary for confirmation [26].

Given the surge in de novo ITP cases and pre-existing ITP exacerbations after COVID-19 vaccination, and the rise in positive platelet-antibody tests since January–June 2021 (Table S3), it remains plausible that the clinically suspected non-HIT-like VITT patients testing positive for platelet-antibodies in our cohort were ultimately diagnosed with ITP [27,28,29,30]. ITP cases have not only been described after vaccination with COVID-19 vaccines (1.13 per 100,000 ChAdOx1 nCoV-19 doses; 0.80 cases of thrombocytopenia per million doses of both BNT162b2 and mRNA-1273), but also after other vaccinations including for hepatitis A, varicella, and measles–mumps–rubella vaccines (1–4 cases per 100,000 MMR doses) [27,31,32,33,34]. Although virus vaccine components and virus-induced molecular mimicry have been mentioned as potential causes for vaccine-induced ITP, it is unclear what triggers the formation of platelet GP-specific antibodies upon vaccination with COVID-19 and other vaccines.

Reports of ITP occurring after infection with COVID-19 [35,36] lead us to investigate fluctuations in ITP reference testing in our laboratory, in order to clarify whether COVID-19 vaccine administration may have contributed to the increase in positive ITP reference tests. Starting in June 2020, the Dutch ITP guideline required testing for platelet-autoantibodies in the MAIPA to support an ITP diagnosis [37], which likely resulted in an increase in platelet-autoantibody tests in the second half of 2020. Requests for platelet-autoantibody tests continued to increase in the following years, which is most likely related to the start of the COVID-19 vaccination strategy in January 2021 and the concomitant clinical awareness for serious adverse effects [27,28,38]. Although the increase in confirmed COVID-19 infections in January/February 2022 [39] appears to coincide with the continuous increase of positive platelet-autoantibody tests, more data on whether the patients in our cohort experienced COVID-19 infections need to be investigated in subsequent studies.

## 5. Conclusions

We tested 232 clinically suspected VITT patients, of whom seven were confirmed HIT-like VITT patients, for the presence of platelet-antibodies. We found 44 patients tested positive for platelet-antibodies, of which none were confirmed HIT-like VITT patients. Therefore, the role of anti-platelet GPs in HIT-like and anti-PF4 mediated VITT appears unlikely. Although further investigation is needed, the presence of platelet-antibodies seemed primarily associated with the occurrence of thrombocytopenia, indicating a potential mechanism of antibody-mediated platelet clearance not directly linked to the development of VITT. Investigating a possible connection between the administered (type of) vaccine(s) and the presence of platelet-antibodies, we found no significant correlation. Similarly, our analysis comparing platelet-antibody profiles of suspected ITP patients to those of suspected VITT patients showed no overall distinctions. In addition, analysis of TPO levels showed the majority of patients with platelet-antibodies and thrombocytopenia had normal TPO levels which could be indicative of ITP, and analysis of ITP reference test requests revealed an increase since the start of the COVID-19 vaccination strategy. Taken together, it is possible that thrombocytopenic patients testing positive for platelet-antibodies who were suspected of having VITT, are de novo or pre-existing ITP patients. However, as ITP is a diagnosis of exclusion and we lack data on pre-existing conditions we cannot conclusively say the patients testing positive for platelet-antibodies are ITP patients. New studies with better clinically defined patients and longitudinal analysis of the presence of platelet-antibodies could reveal more about the presence of platelet-antibodies after COVID-19 vaccination. Overall, more research into the pathophysiological mechanisms of VITT is highly warranted for strengthening diagnostic approaches and identifying therapeutic targets.

## Figures and Tables

**Table 1:** Baseline and clinical characteristics of 232 VITT-suspected patients who were tested in the indirect MAIPA.

	Clinically Suspected Patients (*n* = 232)	Positive for Platelet Antibodies (*n* = 44)	Negative for Platelet Antibodies (*n* = 188)
Demographics			
Median age (IQR)	62 (53–68)	62 (54–69)	60 (53–69)
Female sex (no.(%))	111 (48%)	24 (55%)	87 (46%)
Male sex (no.(%))	121 (53%)	20 (45%)	101 (54%)
Vaccination			
Vaccine type (no.(%))			
Adenoviral vector vaccines	119 (51%)	20 (45%)	99 (53%)
ChAdOx1 nCoV-19	112 (48%)	19 (43%)	93 (50%)
Ad26.COV2.S	7 (3%)	1 (2%)	6 (3%)
mRNA vaccines	113 (49%)	24 (55%)	89 (47%)
mRNA-1273	34 (15%)	7 (16%)	27 (14%)
BTN162b2	79 (34%)	17 (39%)	62 (33%)
Days between admission and vaccination			
Mean (IQR)	21 (8–28)	24 (9–29)	21 (8–28)
Number of vaccination (no.(%))			
First dose	37 (16%)	10 (23%)	31 (17%)
Second dose	68 (29%)	15 (34%)	55 (29%)
Third dose	2 (1%)	-	2 (1%)
No information on dose	125 (54%)	19 (43%)	100 (53%)
Clinical characteristics (no.(%))			
Thrombocytopenia (<100 × 10^9^/L)	151 (65%)	34 (77%)	117 (62%)
Median platelet count (IQR)	51 (18–99)	35 (8–63)	55 (21–108)
No thrombocytopenia	55 (24%)	4 (9%)	51 (27%)
No data on platelet count	26 (11%)	6 (14%)	20 (11%)
Thrombosis	71 (31%)	7 (16%)	64 (34%)
No thrombosis	129 (56%)	31 (71%)	98 (52%)
No data on thrombosis available	32 (14%)	6 (14%)	26 (14%)
Thrombocytopenia and thrombosis	32 (14%)	3 (7%)	29 (15%)
Thrombocytopenia only	119 (51%)	31 (71%)	88 (47%)
Thrombosis only	39 (17%)	4 (9%)	35 (19%)
Neither thrombocytopenia nor thrombosis	19 (8%)	1 (2%)	18 (10%)
No data on both thrombocytopenia and thrombosis	23 (10%)	5 (11%)	18 (10%)
Laboratory tests			
Anti-PF4 ELISA negative (OD < 1.0)	212 (91%)	42 (96%)	170 (90%)
PIPAA negative	206 (97%)	38 (90%)	168 (99%)
PIPAA positive	6 (3%)	4 (10%)	2 (1%)
Anti-PF4 ELISA weak-positive (1.0 ≤ OD < 2.0)	7 (3%)	1 (2%)	6 (3%)
PIPAA negative	6 (86%)	1 (100%)	5 (83%)
PIPAA positive	1 (14%)	-	1 (17%)
Anti-PF4 ELISA positive (OD ≥ 2.0)	13 (6%)	1 (2%)	12 (6%)
PIPAA negative	3 (23%)	1 (100%)	2 (17%)
PIPAA positive	10 (77%)	-	10 (83%)

## Data Availability

The data that support the findings of this study are available from the corresponding author, r.kapur@sanquin.nl, upon reasonable request.

## Supplementary Materials

The following supporting information can be downloaded at: https://www.mdpi.com/article/10.3390/antib13020035/s1, Figure S1: Analysis of TPO levels of 42 platelet-antibody-positive patients and of 178 platelet-antibody-negative patients; Table S1: Details HIT-like VITT patients tested for platelet-antibodies; Table S2: Results from anti-PF4 IgG ELISA, FcγRIIa-dependent PIPAA and indirect MAIPA. Table S3: Requests for ITP diagnostic reference testing.
