# Supplementary material for: Antibodies against Platelet Glycoproteins in Clinically Suspected VITT Patients
Source: Antibodies (Basel). 2024 May 1;13(2):35. doi: 10.3390/antib13020035 (PMC11130846; doi:10.3390/antib13020035)
Supplement: Supplementary file 1 [file antibodies-13-00035-s001.zip › antibodies-2864191-supplementary.pdf]

SUPPLEMENTAL FIGURE LEGENDS

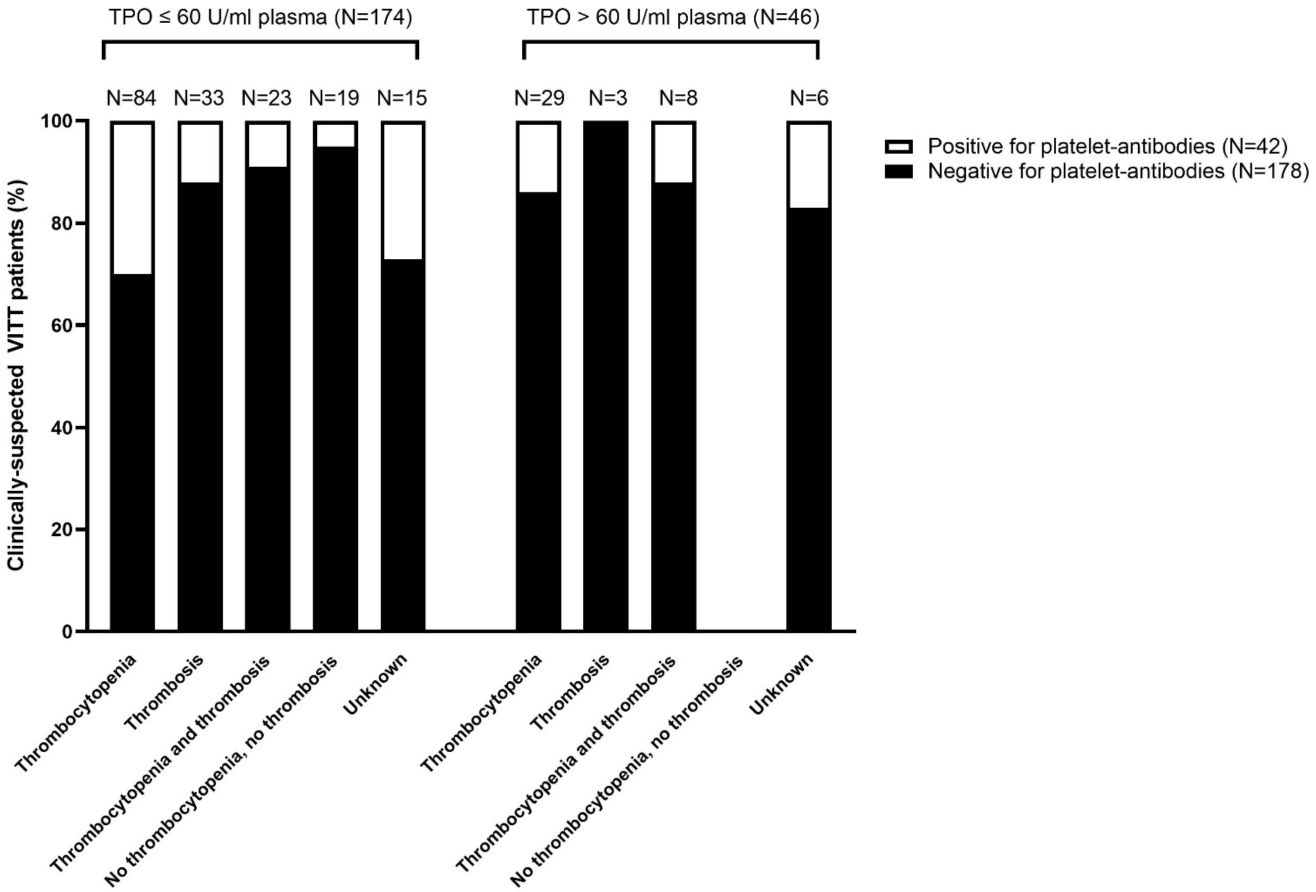


Figure S1: Analysis of TPO levels of 42 platelet-antibody positive patients and of 178 platelet-antibody negative patients. Out of the 42 platelet-antibody positive patients 36 patients had normal TPO levels and six patients had elevated TPO levels. Of the patients that tested negative for platelet-antibodies: 138 patients had normal TPO levels and 40 patients had elevated TPO levels.

SUPPLEMENTAL TABLES

Supplemental Table S1: Details HIT-like VITT patients tested for platelet-antibodies. Patients who were considered HIT-like VITT patients (with thrombocytopenia and thrombosis) and were tested for the presence of platelet-antibodies in the indirect MAIPA. *Patient 2 was tested at 20 days after vaccination but was not considered probable VITT after a negative FcγRIIa-dependent PIPAA at 22 days after vaccination, the second FcγRIIa-dependent PIPAA at 42 days after vaccination was positive.

| Patient | Sex (M/F) | Age | Vaccine | Dose | Days since vaccination | anti-PF4 ELISA (OD) | FcγRIIa-dependent PIPAA(+/-) | Platelet count at admission | Site thrombosis | Indirect MAIPA (+/-) | TPO levels |
|---|---|---|---|---|---|---|---|---|---|---|---|
| 1 | M | 67 | ChAdOx1 nCov-19 | - | 12 | >3.0 | + | <100 | CVST | - | 28 |
| 2* | F | 32 | ChAdOx1 nCov-19 | - | 42 | 2.1 | + | <100 | Mesenteric thrombosis | - | 24 |
| 3 | F | 75 | ChAdOx1 nCov-19 | - | - | >3.0 | + | <100 | DVT | - | 67 (elevated) |
| 4 | M | 64 | ChAdOx1 nCov-19 | 1 | 9 | >3.0 | + | <100 | Intracranial | - | 115 (elevated) |
| 5 | M | 61 | ChAdOx1 nCov-19 | - | - | >3.0 | + | <100 | Multiple | - | 14 |
| 6 | F | 28 | Ad26.COV2.S | 1 | 11 | 2.24 | + | <100 | CVST | - | 22 |
| 7 | F | 53 | Ad26.COV2.S | 1 | - | >3.0 | + | <100 | Other | - | 15 |

Supplemental Table S2: Results from anti-PF4 IgG ELISA, FcγRIIa-dependent PIPAA and indirect MAIPA. * considered HIT-like VITT patients. † tested positive in both the anti-PF4 IgG ELISA and PIPAA but did not have thrombocytopenia and thrombosis or clinical characteristics were unknown.

| **Clinical characteristics** | **anti-PF4 IgG ELISA** | **FcγRIIa-dependent PIPAA (pos/neg)** | **Indirect MAIPA (pos/neg)** |
|---|---|---|---|
| Thrombocytopenia only (*n*=119) | Neg (*n*=111) | Neg (*n*=108) | Neg (*n*=79) |
| | | | Pos (*n*=29) |
| | | Pos (*n*=3) | Neg (*n*=1) |
| | | | Pos (*n*=2) |
| | Pos (*n*=8) | Neg (*n*=5) | Neg (*n*=5) |
| | | Pos (*n*=3)† | Neg (*n*=3) |
| Thrombosis only (*n*=39) | Neg (*n*=38) | Neg (*n*=36) | Neg (*n*=33) |
| | | | Pos (*n*=3) |
| | | Pos (*n*=2) | Neg (*n*=1) |
| | | | Pos (*n*=1) |
| | Pos (*n*=1) | Pos (*n*=1)† | Neg (*n*=1) |
| Thrombocytopenia and thrombosis (*n*=32) | Neg (*n*=22) | Neg (*n*=21) | Neg (*n*=20) |
| | | | Pos (*n*=1) |
| | | Pos (*n*=1) | Pos (*n*=1) |
| | Pos (*n*=10) | Neg (*n*=3) | Neg (*n*=2) |
| | | | Pos (*n*=1) |
| | | Pos (*n*=7)* | Neg (*n*=7) |
| Clinical characteristics unknown (*n*=23) | Neg (*n*=22) | Neg (*n*=22) | Neg (*n*=18) |
| | | | Pos (*n*=4) |
| | | | Pos (*n*=1) |
| No thrombocytopenia, no thrombosis (*n*=19) | Neg (*n*=19) | Neg (*n*=19) | Neg (*n*=18) |
| | | | Pos (*n*=1) |

Supplemental Table S3: Requests for ITP diagnostic reference testing. Increase in platelet-autoantibody testing and positive tests after implementation of the ITP guidelines in June 2020 and a further increase in testing and positive tests since the start of the vaccination on January 8, 2021.

| **Time period** | **Patients tested positive for platelet-autoantibodies of total number of tests performed N(%)** |
|---|---|
| July-December 2019 | 132 (42.6) |
| January-June 2020 | 128 (46.9) |
| July – December 2020 | 166 (38.1) |
| January – June 2021 | 240 (44.6) |
| July-December 2021 | 228 (42.0) |
| January -June 2022 | 273 (48.3) |
